# Beyond height and weight: a programme of school nurse assessed skinfold measurements from white British and South Asian origin children aged 4–5 years within the Born in Bradford cohort study

**DOI:** 10.1136/bmjopen-2015-008630

**Published:** 2015-11-26

**Authors:** Jane West, Gillian Santorelli, Laura Lennon, Kathy O'Connell, John Corkett, John Wright, Shirley Brierley, Peter Whincup, Noel Cameron, Debbie A Lawlor

**Affiliations:** 1 Bradford Institute for Health Research, Bradford Royal Infirmary, Bradford, UK; 2 School of Social and Community Medicine, University of Bristol, Bristol, UK; 3 Leeds Institute of Clinical Trials Research, University of Leeds, Leeds, UK; 4 Department of Public Health, Bradford District Metropolitan Council, Bradford, UK; 5 Population Health Research Institute, St George's, University of London, London, UK; 6 School of Sport, Exercise and Health Sciences, Loughborough University, Loughborough, UK; 7 MRC Integrative Epidemiology Unit, University of Bristol, Bristol, UK

**Keywords:** PREVENTIVE MEDICINE

## Abstract

**Objective:**

To describe the feasibility, reliability and additional information gained from collecting additional body fatness measures (beyond height and weight) from UK reception year children.

**Design:**

Prospective cohort study.

**Setting:**

Bradford, UK.

**Participants:**

2458 reception year children participating in the Born in Bradford (BiB) cohort study.

**Main outcome measures:**

The feasibility and reliability of subscapular and triceps skinfold measurements and differences in adiposity between ethnic groups.

**Results:**

Of those children who were matched to their school, 91% had a subscapular skinfold measurement and 92% had a triceps skinfold measurement recorded. Reliability was generally over 90% for all measurers and both measurements. Pakistani children were slightly taller but weighed less and had lower triceps skinfold thickness (mean difference −1.8 mm, 95% CI −2.1 to −1.4 mm) but higher subscapular (mean difference 0.1 mm, 95% CI −0.1 to 0.4 mm) than white British children.

**Conclusions:**

We have shown that it is feasible for school nurses to collect skinfold measurements in a similar way to the height and weight measurements collected from reception year children for the National Child Measurement Programme (NCMP), and that these measurements are reliable. It is important for healthcare practice to acknowledge ethnic-specific risk and these additional measurements can provide important information to examine population-level risk in populations with large proportions of South Asian children.

**Strengths and limitations of this study**

- A key strength of this study is that we report for the first time, a city-wide programme of child skinfold thickness measurements by school nurse teams that compliment routine height and weight measurements to estimate fatness in reception age children.
- These teams successfully achieved coverage similar to that reported for the National Child Measurement Programme (NCMP) and our results suggest that these measurements are reliable.
- A potential limitation is that we were unable to match 33% of study children to their primary school, but we anticipate that for school years beyond reception, linkage of at least 85% of Born in Bradford (BiB) children to their school will be achieved.

## Background

Childhood obesity is likely to play an important role in future cardiometabolic health^1^ and its prevention is now a global public health priority.^2^ Despite recent reports that rates of childhood obesity are stabilising,^3^ overall prevalence remains high ranging from a fifth to a third of young children in the USA and UK now overweight or obese.^4^ ^5^ Concerns about childhood obesity have resulted in national or regional surveillance programmes that record weight and height in order to monitor trends in overweight and obesity based on body mass index (BMI), for example, the National Health and Nutrition Examination Survey (NHANES) in the USA and the National Child Measurement Programme (NCMP) in England. While BMI in childhood and adolescence is robustly associated with markers of cardiometabolic health and future incidence of type 2 diabetes and cardiovascular disease in adults,^6–8^ it may not be able to identify ethnic differences in either the levels or distribution of adiposity and hence distinguish risk between different ethnic groups at a population level. There is increasing evidence that for an equivalent BMI, South Asian children have greater total and central adiposity than white British children^9^ ^10^ and are potentially more metabolically sensitive to body fat.^11^ Our own work using the Born in Bradford (BiB) cohort (used in this publication)^12–14^ and that of others,^15–18^ suggests that even at birth South Asian infants might have greater levels of fat for a given birth weight than white Europeans. Thus, public health surveillance that monitors overweight and obesity levels in children based on BMI might not accurately reflect future health burden. One solution might be to lower BMI thresholds for defining overweight and obesity in South Asian children; however, lowering such thresholds in South Asian adults does not seem to better predict future risk^19^ ^20^ and therefore is unlikely to do so in children. An alternative would be to add additional measurements of body fat and distribution that might be able to provide a better idea of population-level risk in populations with large proportions of South Asian children.

Our aim in this paper is to examine the feasibility of adding reliable measurements of subscapular and triceps skinfold thickness to the UK NCMP in one city with a large proportion of South Asian children. We have chosen subscapular and triceps skinfolds because these do not require expensive and difficult to transport equipment, do not require children to show their waist, which some may find embarrassing or invasive in the school setting, and because these measures have been shown to accurately reflect peripheral and central adiposity levels.^15^ ^21^ Furthermore, other studies that include skinfold measurements obtained by research staff show that they are better discriminators of ethnic differences than is BMI.^9^ ^22^

Specific objectives were (1) to examining the feasibility of school nurse teams undertaking skinfold thickness measurements from reception age children (4–5 years), as determined by the proportions of children in the cohort and by ethnicity and age who have valid skinfold measurements and how these compare to proportions with valid weight and height measurements, (2) to examine inter-rater reliability by comparing measurements undertaken on the same child between each nurse and a trained researcher in a subgroup of participants and (3) to compare BMI and the additional information gained from skinfold measurements between ethnic groups.

## Methods

### Participants

This study was nested in the BiB cohort which is a prospective pregnancy and birth cohort based in the sixth largest city in the UK: Bradford in the North of England. The study was established to examine how genetic, nutritional, environmental, behavioural and social factors impact on health and development during childhood, and subsequently adult life, in a deprived multiethnic population. The study recruited women during pregnancy and has followed them, their partners and their infants through childhood. To be eligible for the study women had to attend antenatal booking clinic between March 2007 and December 2010 and be booked to give birth in the city of Bradford. Full details of the study methodology have been previously reported.^23^ A total of 12 453 women who gave birth to 13 818 liveborn children were recruited to the study. Of these 13 818 children, 3730 were eligible to start school in the academic year 2012/2013 and these 3730 children form the sample for this study. Parents of eligible children were mailed information about the skinfold measurements 8 weeks prior to the scheduled measurements with an ‘opt out’ consent form and prepaid envelope should they wish to withdraw their child from the measurements. The opt-out consent is consistent with the consent process used for the NCMP and was approved by the National Health Service research ethics committee. Parental consent was refused for 77 (2%) of children with similar proportions in the two main ethnic groups (white British 36 (47%) and South Asian 41 (53%)) and of the 3653 for whom consent was not declined 1195 (33%) could not be matched to their school, again this did not vary markedly by ethnicity (538 (45%) were of South Asian origin and 657 (55%) white British). The remaining 2458 form the sample for this study. Ethics approval for the study was granted by Bradford National Health Service Research Ethics Committee (ref 06/Q1202/48).

### Assessment of ethnicity

Ethnicity was self-reported at the mother's questionnaire interview and based on UK Office of National Statistics guidance details of which have been previously reported.^14^ For the purpose of this study, the children are defined as white British; other white; Pakistani; other South Asian; other ethnicity.

### Linkage of BiB participants to primary schools

The linkage to education data was done using a deterministic match based on the first name, last name, gender, date of birth and postcode. The rule for the matching was that there has to be a single definite match on each of these attributes in the Local Education Authority (LEA) database for them to provide us with the unique pupil number (UPN). Once we had the UPN we could download data about the children, including their school from the LEA website portal and verify for ourselves that the matches were accurate.

### The Bradford school nursing service

The school nursing service in Bradford includes 110 nurses, nursery nurses and healthcare support workers working across 11 teams and covering 143 primary schools in Bradford. Each school has a named school nurse which ensures an integrated service where the school nurse and team are accessible and well known to the education staff, parents and children within each school. This relationship has supported a high response for the NCMP with 88–91% of reception age children in the city having valid height and weight measurements over the three academic years 2010/2011–2012/2013.

### Training of school nurses and completion of anthropometric measurements

Following initial discussions with the local authority and school nurses about adding skinfold measurements to the NCMP weight and height measurements, it was agreed that the skinfold measurements would be collected at a separate school visit from those undertaken to collect height and weight for the NCMP. This was to allow time for matching of BiB children to school reception class lists that for many schools are not completed and submitted to the local authority until the spring term. All of the skinfold measurements were conducted within 4 months of the NCMP height and weight measures (mean time since NCMP height and weight was 13.1 weeks (SD 7.5)). School nurses/health support workers (for ease of reading hereafter referred to as ‘school nurses’) in Bradford have all been trained in completing height and weight measurements for the NCMP by the Child Growth Foundation and receive yearly measurement training updates. Height was measured using the Leicester Height Measure (Seca Ltd, Leicester, UK) and weight using Seca digital scales (Seca Ltd, Leicester, UK). All height and weight measurements were recorded to one decimal place (ie, to the nearest mm for height and the nearest 0.1 kg for weight).

Experienced trained research staff from the BiB study trained the school nurses in how to measure triceps and subscapular skinfolds in children. Training was provided at regular intervals and the school nurses were provided with a written protocol (the same as that used previously for BiB measurements) for measuring skinfolds. Periodic monitoring and updating of training by the BiB researchers continued throughout the school year during which the nurses were collecting these data. Skinfold measurements were collected using Holtain Tanner/Whitehouse Calipers (Holtain Ltd) on the left side of the body. Children were seated and removed their left arm only from clothing.

### Assessment of feasibility and reliability of skinfold measurement

We assessed feasibility by recording the number and percentage of children who had parental consent, child assent and valid measurements of each skinfold measurement and compared these with the number and percentage of children with NCMP height and weight measurements. These comparisons were made for the whole study sample (for school year 2012/2013) and also stratified by (1) ethnicity (defined as white British; other white; Pakistani; other South Asian; other ethnicity) and (2) gender. Feasibility would be supported by having similar proportions of children with valid skinfold measurements to the proportion with height and weight measurements.

The 2458 children in this study attended 135 Bradford primary schools. Height and weight measures for the NCMP were collected by 20 school nurses and 7 of these also collected the BiB skinfold measurements reported here. We assessed interobserver reliability in a subgroup of 84 children (12 children for each of the 7 school nurses) in whom the measurements were repeated by a trained assessor (JW) who was blind to the initial measurement.

## Statistical analyses

All analyses were performed using STATA/SE software (Stata/SE V.12 for Windows, StataCorp LP, College Station, Texas, USA). The proportion (expressed as a percentage) of children with skinfold measurements was compared with the proportion of those with NCMP height and weight measurements stratified by ethnicity and by gender.

To assess reliability, we used the absolute technical error of measurement (TEM), the relative TEM and the coefficient of reliability (R). These three methods assess different aspects of reliability such that the TEM provides an indication of how repeat measurements vary from the mean, the relative TEM provides a measure of size of error (variation from the mean) in relation to the magnitude of the mean and R provides the proportion of variation between measurements. The WHO suggest that where an expert assessor is available, acceptable TEM cut-offs should be based on the expert's intraobserver TEM where TEM values for other observers in the study should then lie within ±2 times the expert's TEM. Where an expert is not available, the average of well-trained observers can be used to set acceptable limits^24^ (shown in the final column of table 2). We took the latter approach here (using inter-reliability TEM), since among the nurses there was no individual who could be considered more experienced than the others and intrareliability repeat measurements were not taken in this study and were therefore not available for the trained assessor (JW). In addition, we used Bland-Altman plots to examine agreement between the measurers and trained researcher to identify any systematic bias, for example, differences being greater or smaller depending on the overall mean of the two measurements.

Unadjusted means of height, weight, BMI, subscapular and triceps skinfold thickness are presented by ethnic group in the original scales of each measurement (m, kg, kg/m^2^ and mm, respectively, for height, weight, BMI and skinfolds). These allow differences in meaningful units to be observed. To explore whether any ethnic differences had different magnitudes between the different anthropometric measurements, we also generated age and sex internally standardised z-scores for each measurement and regressed these z-scores on ethnicity.

## Results

### Feasibility

Of the 2458 BiB children in reception classes for the academic year 2012/2013 who were successfully linked to their school, 91% had a subscapular skinfold measurement and 92% had a triceps skinfold measurement taken. Height and weight were recorded at separate visits as part of the NCMP and were available for 91% and 93% of the children in this sample, respectively (table 1). There were no notable ethnic or sex differences in the proportions completing the four measurements.

**Table 1:** Number (%) of children with height, weight and skinfold thickness measurements by ethnic group and gender

	Height	Weight	Triceps skinfold	Subscapular skinfold
Whole sample (N=2458)	2242 (91.2)	2284 (92.9)	2270 (92.4)	2243 (91.3)
Ethnic group				
White British (N=785)	731 (93.1)	736 (93.8)	719 (91.6)	712 (90.7)
Other White (N=31)	28 (90.3)	29 (93.6)	29 (93.6)	29 (93.6)
Pakistani (N=904)	819 (90.6)	844 (93.4)	844 (93.4)	830 (91.8)
Other South Asian (N=97)	92 (94.9)	91 (93.8)	92 (94.9)	91 (93.8)
Other (N=114)	102 (89.5)	103 (90.4)	103 (90.4)	102 (89.5)
Ethnic group missing (N=527)	470 (89.2)	481 (91.3)	483 (91.7)	479 (90.9)
Gender				
Male (N=1185)	1068 (90.1)	1084 (91.5)	1088 (91.8)	1074 (90.6)
Female (N=1273)	1174 (92.2)	1200 (94.3)	1182 (92.9)	1169 (91.8)

### Reliability

Results of interobserver reliability assessments are presented in table 2. The TEM values were all within ±2 times the average for all assessors. Relative TEM values ranged from 2.24% to 6.19% for triceps measurements and from 3.88% to 10.57% for subscapular measurements. R was generally over 90% for all measurers other than measurer 2 (triceps skinfold result 88.7%; subscapular skinfold result 87.5%) and one measurer 6 (subscapular skinfold result 81.6%). Bland-Altman plots (see online supplementary figures S1–S14) showed that all mean differences were close to zero and were spread evenly across either side of the line suggesting no systematic bias.

**Table 2:** Technical errors of measurement (TEM) and reliability values for inter-reliability assessments*

Measurer	Absolute TEM (mm)	Relative TEM (%)	R (%)	Average TEM All assessors (mm)
Measurer 1				
Triceps	0.34	3.04	99.3	0.43
Subscapular	0.86	10.57	97.2	0.57
Measurer 2				
Triceps	0.61	6.19	88.7	0.43
Subscapular	0.60	9.40	87.5	0.57
Measurer 3				
Triceps	0.53	5.22	97.6	0.43
Subscapular	0.42	5.66	98.2	0.57
Measurer 4				
Triceps	0.29	2.47	99.2	0.43
Subscapular	0.28	3.88	99.1	0.57
Measurer 5				
Triceps	0.28	2.24	99.4	0.43
Subscapular	0.63	7.88	98.4	0.57
Measurer 6				
Triceps	0.43	5.45	95.0	0.43
Subscapular	0.63	10.30	81.6	0.57
Measurer 7				
Triceps	0.64	5.72	95.3	0.43
Subscapular	0.59	8.87	92.4	0.57

*Measurements taken by the school nurse and repeated by a trained assessor. Results based on 12 replicate measurements for each school nurse.

### Ethnic variations in height, weight, BMI and skinfolds

Differences in height, weight, BMI, subscapular and triceps skinfold thickness between ethnic groups and mean differences for each ethnic group relative to white British children are presented in table 3. Children classified as having other white ethnicity had similar measurements to white British children but weighed slightly more and had lower triceps skinfold thickness. Pakistani children were slightly taller but weighed less and had lower triceps skinfold thickness than white British children. A similar pattern was seen for children of other South Asian origin. Those of other ethnicity and those where ethnicity information was missing, had similar height and subscapular skinfold thickness but weighed less and had lower triceps skinfold thickness than white British children. There were no marked differences in age or gender between ethnic groups.

**Table 3:** Means (SD) for each measurement by ethnic group and unadjusted mean differences in measurements for each ethnic group relative to white British children

Ethnic group	Height (m)	Weight (kg)	Triceps skinfold (mm)	Subscapular skinfold (mm)	BMI
Whole sample N=2458					
Mean (SD)	1.08 (0.05)	19.0 (3.1)	10.1 (3.4)	6.3 (2.2)	16.1 (1.8)
White British N=785					
Mean (SD)	1.08 (0.05)	19.2 (2.9)	11.1 (3.2)	6.2 (1.9)	16.3 (1.6)
White other N=31					
Mean (SD)	1.09 (0.05)	19.6 (2.6)	10.4 (3.0)	6.3 (2.0)	16.4 (1.3)
Mean difference*	0.01 (−0.01, 0.02)	0.4 (−0.7, 1.6)	−0.8 (−2.0, 0.5)	0.1 (−0.7, 0.9)	0.02 (−0.65, 0.68)
Pakistani N=904					
Mean (SD)	1.09 (0.05)	18.9 (3.3)	9.4 (3.5)	6.3 (2.4)	15.9 (1.9)
Mean difference*	0.01 (0.00, 0.01)	−0.3 (−0.6, 0.0)	−1.8 (−2.1, −1.4)	0.1 (−0.1, 0.4)	−0.46 (−0.64, −0.28)
South Asian other† N=97					
Mean (SD)	1.07 (0.05)	18.4 (3.6)	10.3 (3.8)	6.4 (2.4)	15.9 (2.1)
Mean difference*	−0.01 (−0.02, 0.00)	−0.8 (−1.4, −0.1)	−0.9 (−1.6, −0.1)	0.2 (−0.3, 0.7)	−0.45 (−0.84, −0.06)
Other ethnicity‡ N=114					
Mean (SD)	1.08 (0.05)	18.7 (3.0)	9.8 (2.8)	6.3 (2.0)	16.2 (1.7)
Mean difference*	−0.01 (−0.02, 0.01)	−0.4 (−1.1, 0.2)	−1.4 (−2.1, −0.7)	0.1 (−0.4, 0.5)	−0.19 (−0.55, 0.18)
Ethnic group missing N=52					
Mean (SD)	1.08 (0.05)	18.9 (3.2)	9.9 (3.3)	6.3 (2.2)	16.1 (1.8)
Mean difference*	0.00 (−0.01, 0.01)	−0.2 (−0.6, 0.1)	−1.2 (−1.6, −0.8)	0.1 (−0.2, 0.4)	−0.23 (−0.44, −0.02)

*Unadjusted mean difference (95% CI) relative to white British children.

†Indian; Bangladeshi.

‡Black; mixed white and black; mixed white and South Asian.

BMI, body mass index.

**Figure 1:**
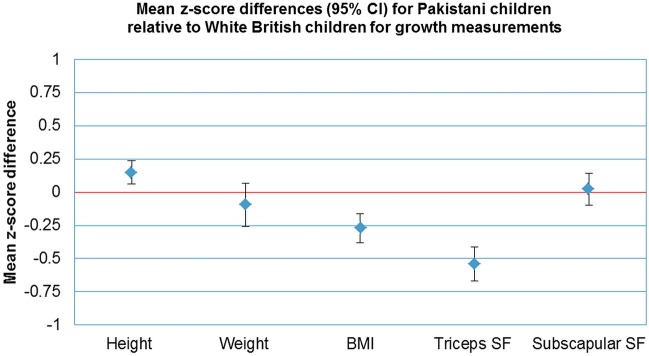
Mean z-score differences (95% CI) for Pakistani infants relative to white British infants (BMI, body mass index; SF, skinfold).

Figure 1 shows mean z-score differences in height, weight, BMI, triceps and subscapular skinfold for Pakistani children relative to white British children (the 2 largest groups in this sample). Compared with white British children, the Pakistani children were taller, but weighed less with the magnitudes of these differences being similar to each other. This resulted in notably lower BMI in Pakistani, compared with white British children of −0.27 SDs (95% CI −0.37 to −0.17). Triceps skinfold was also markedly lower in Pakistani compared with white British children, but subscapular skinfolds, while not statistically significant, appeared similar in both groups (mean difference 0.1 mm, 95% CI 0.1 to 0.4 mm). When we further adjusted for BMI (data not presented), triceps skinfolds were 0.20 SD lower in Pakistani compared with white British children (95% CI 0.25 to −0.16) and subscapular skinfolds were 0.10 SD higher (95% CI 0.06 to 0.14).

## Discussion

Evidence increasingly suggests that South Asians have a characteristic phenotype of proportionately greater adiposity, increased insulin resistance and an increased risk of cardiometabolic disease compared with white Europeans.^9^ ^25^ Using data from the BiB cohort (as used here), we have previously shown that similar ethnic differences appear to be present at birth.^12^ Specifically, we showed that, despite being born smaller and lighter, for a given birth weight Pakistani infants had similar triceps and subscapular skinfold thickness measurements to white British infants suggesting greater relative fatness; they also had higher cord leptin levels even without taking birth weight into account.^12^ ^14^ In light of this, and in the knowledge that this greater fatness may underlie the increased risk of type 2 diabetes among South Asian populations,^26^ our aim was to develop a programme of growth monitoring to compliment routine height and weight assessments, that would be feasible to implement by school nurses but that could provide estimates of body fat that might better characterise ethnic differences in risk (ie, triceps and subscapular skinfold thickness). In this study, we have shown that it is feasible to implement such a programme and that similar high proportions of children having valid measurements of subscapular and triceps skinfolds, as found for weight and height measurements can be obtained. Our results show that school nurses can collect skinfold measurements with acceptable levels of reliability and with no evidence of systematic bias relative to a trained assessor.

A number of previous studies have successfully collected skinfold thickness measurements from UK children either in school using trained research staff^10^ or at research clinics,^27^ whereas here we have shown the feasibility of school nurses reliably collecting these data. As such, we have shown that it is feasible to collect these additional measurements within the existing routine programme used to assess childhood height and weight in the UK (the NCMP).

We have previously shown skinfold measurements can be reliably recorded at birth by research administrators.^28^ In that report, we also drew attention to the inconsistencies in the methods used and in the interpretation of results across different studies. Here, in the continued absence of any universal consensus regarding the most appropriate statistical method,^29–32^ we have again used three commonly reported measurements that assess different aspects of reliability. Our TEM results indicated all measurements were reliable, relative TEM was below 11% and R was above 90% for most measurements among most staff. These results show better reliability compared with our birth assessments particularly in terms of higher R and generally lower relative TEM, but as we found previously with birth measurements, the three methods did not always rank the measurers in the same order underlining the importance of using more than one approach.^28^ Subscapular measurements were slightly less reliable than triceps which may be due to difficulties obtaining the measurement with only one arm removed from clothing.

In our previous publications using this cohort, we have shown that Pakistani compared with white British infants have markedly lower birth weight. They also had thinner birth subscapular and triceps skinfold thicknesses, but the magnitudes of these differences were smaller than those seen for birth weight. Now at age 4–5 years, the Pakistani children remain lighter, but they are now taller, which together contribute to a lower BMI than white British children. However, there are now notable differences in the two skinfold thicknesses, with triceps being lower in the Pakistani children but subscapular being the same in Pakistani and white British children. As Pakistani children were lighter and had on average a lower BMI, one might expect a lower, rather than similar subscapular skinfold measurement. For consistency with existing evidence and results reported at birth in this cohort,^12^ we further adjusted for BMI, the difference in triceps skinfold reduced but the difference in subscapular skinfold further increased. Since triceps skinfolds are considered to represent peripheral fat and subscapular centrally distributed fat, these preliminary findings suggest that the Pakistani children have begun to develop markedly more centrally distributed adiposity than white British children. As Pakistani children also had lower BMI, our results highlight that relying solely on BMI in routine surveys of childhood growth and well-being might incorrectly suggest some populations (here Pakistani) are healthier than others (white British) when the opposite might be true. Our findings, based here on school nurse assessments, are consistent with previous studies of UK schoolchildren assessed under researcher conditions, including the Child Health and Heart Study in England (CHASE) which found higher levels of adiposity based on skinfold and bioimpedance assessments, among South Asian children aged 9–11 years but that BMI underestimated the magnitude of the higher adiposity in South Asian children at this age.^9^

Ethnic differences in adiposity and its distribution have been reported consistently in adults and are now being identified in infants and children.^9^ ^12^ ^16^ On the basis of these findings it could be suggested that lower BMI thresholds are used to indicate overweight and obesity in South Asian children, but this has been widely debated in relation to South Asian adults^33^ ^34^ and there remains no clear threshold at which BMI results in adverse health outcomes.^20^ ^35^ ^36^ Importantly, BMI cannot determine adiposity distribution. While waist circumference, waist:hip ratio or waist:height ratio have all been proposed to identify centrally distributed adiposity and identify those at risk, evidence from children and adults suggests a very strong correlation with BMI and similar magnitudes of association with cardiometabolic risk for these measurements as that seen with BMI.^9^ ^37–39^ Further, many children might be reluctant to uncover to the extent required for a waist measurement.

To our knowledge, this is the first UK study to undertake a city-wide programme of child skinfold measures that can compliment routine height and weight measurements to estimate fatness in reception age children. School nurse teams successfully achieved coverage similar to that reported for the NCMP^4^ height and weight measurements.

A limitation of our study was that we were unable to link 33% of BiB children to their primary school. This was mostly due to delays in schools submitting reception year pupil information to the local authority and in a minority of cases resulted from children attending schools outside the Bradford local authority area. Since completing this work we have worked with the nurses, schools and parents and are now preparing to add measurements of blood pressure, bioimpedance and the distribution of accelerometers to assess physical activity, as well as continuing to measure skinfold thicknesses in school children at and beyond reception year. Initial attempts at linking for school years after reception suggest that for those we will achieve linkage of at least 85% of BiB children. Importantly, there were no marked differences by ethnicity in either linkage or completion of the skinfold measurements.

To minimise discomfort and potential distress to the children, which might occur with multiple repeated measurements, we only performed inter-rater assessments. School nurse teams received comprehensive training, all staff were monitored by a trained assessor before undertaking any school visits, ongoing monitoring and training continued throughout the measurement period and all equipment was recalibrated at the start of the academic year. We therefore believe the potential for intraobserver error was minimal. Lastly, we were unable to include other South Asians (Indian and Bangladeshi) as separate groups in our analyses due to relatively small numbers of these populations in the city of Bradford. Our results may not be generalisable to other South Asian groups, though the similarity of our findings to those of CHASE^9^ suggests they are likely to.

## Conclusion

Using early results from the BiB cohort, we have shown it is feasible for school nurses in their role as public health practitioners, to collect skinfold measurements in a similar way to the height and weight measurements collected from reception year children for the NCMP, and that these measurements are reliable. We believe these additional measurements could provide important information to examine population-level risk in populations with large proportions of South Asian children. We acknowledge that further research is required to determine whether these additional measurements do better identify risk of cardiometabolic risk in comparison to BMI and that the cost-effectiveness of these measurements is supportive of their more widespread use.
